# Metabolizable Protein: 2. Requirements for Maintenance in Growing Saanen Goats

**DOI:** 10.3389/fvets.2021.650203

**Published:** 2021-06-07

**Authors:** Anaiane P. Souza, Julián A. C. Vargas, Marcia H. M. R. Fernandes, Amélia K. Almeida, Kleber T. Resende, Izabelle A. M. A. Teixeira

**Affiliations:** ^1^Department of Animal Sciences, Universidade Estadual Paulista, São Paulo, Brazil; ^2^Institute of Studies of the Humid Tropic, Universidade Federal do Sul e Sudeste do Pará, Xinguara, Brazil; ^3^Department of Animal Science, Universidade Federal Rural da Amazônia, Parauapebas, Brazil; ^4^University of New England, Armidale, NSW, Australia

**Keywords:** comparative slaughter, metabolism, N balance, ruminant, sex, efficiency of use, dairy goat

## Abstract

This study aimed to estimate the protein requirements for the maintenance of growing Saanen goats of different sexes from 5 to 45 kg of body weight (BW) using two methods and applying a meta-analysis. For this purpose, two datasets were used. One dataset was used to evaluate the effects of sex on the protein requirements for maintenance using the comparative slaughter technique. This dataset was composed of 185 individual records (80 intact males, 62 castrated males, and 43 females) from six studies. The other dataset was used to evaluate the effects of sex on the protein requirements for maintenance using the N balance method. This dataset was composed of 136 individual records (59 intact males, 43 castrated males, and 34 females) from six studies. All studies applied an experimental design that provided different levels of N intake and different levels of N retention, allowing the development of regression equations to predict the net protein requirement for maintenance (NP_M_) and the metabolizable protein (MP) requirements for maintenance (MP_M_) in Saanen goats. The efficiency of MP use for maintenance (k_PM_) was computed as NP_M_/MP_M_. The efficiency of MP use for gain (k_PG_) was calculated using the equation of daily protein retained against daily MP intake above maintenance. A meta-analysis was applied using the MIXED procedure of SAS, in which sex was considered a fixed effect, and blocks nested in the studies and goat sex were considered as random effects. The NP_M_ did not differ between sexes, irrespective of the approach used. The daily NP_M_ estimated was 1.23 g/kg^0.75^ BW when using the comparative slaughter technique, while it was 3.18 g/kg^0.75^ BW when using the N balance technique for growing Saanen goats. The MP_M_ estimated was 3.8 g/kg^0.75^ BW, the k_PM_ was 0.33, and the k_PG_ was 0.52. We observed that the NP_M_ when using the comparative slaughter technique in growing Saanen goats is lower than that recommended by the current small ruminant feeding systems; on the other hand, the MP_M_ was similar to previous reports by the feeding systems. Sex did not affect the protein requirements for maintenance and the efficiencies of use of metabolizable protein.

## Introduction

Appropriate estimates of the protein requirements for maintenance and growth in goats support the nutritionist's decisions to formulate diets for improving animal production. Besides, inadequate balancing of protein content in ruminant's diet stimulates fecal and urinary nitrogen (N) excretion, which contributes to acid deposition, eutrophication, climate change, and respiratory diseases in humans (1, 2). Hence, accurate information regarding the protein requirements of dairy goats and the factors that affect them is pivotal to accomplish efficient diet formulation from a sustainable standpoint. One of these factors is sex, which impacts the body protein content of growing dairy goats (3).

Protein requirements for maintenance of ruminants include endogenous urinary protein, endogenous fecal protein, and dermal protein losses (4). Based on it, the N balance has been the most commonly used method for measuring the protein losses related to maintenance (5). Another method used for estimating the protein requirements for maintenance has been the comparative slaughter technique, which is based on the differences in body composition of animals slaughtered at different weights and nutritional levels (6–8). Even though retained N, measured by N balance methods or slaughter techniques, should be similar because it refers to a similar concept, the literature suggests that protein requirements for maintenance estimated by N balance studies are greater than those reported by the comparative slaughter studies (5). However, the reasons of these differences have not been conclusive and may be attributed to changes in dietary and environmental conditions across studies. Moreover, it has not been conclusive if sex has influenced these differences as well.

Multiple studies were conducted at our institution to estimate the protein requirements for maintenance in dairy goats of different sexes. We pooled and analyzed them under two meta-analyses for estimating the protein requirements for the maintenance of Saanen goats using the N balance and comparative slaughter methods. Our hypothesis is that sex influences the protein requirements for maintenance, which would be similar regardless of method used. In this sense, this study aimed to estimate the protein requirements for maintenance of growing Saanen goats of different sexes from 5 to 45 kg of body weight (BW) using two different methods and applying a meta-analysis.

## Materials and Methods

### Ethics Statement

All procedures used in the individual studies followed the University's Animal Care Committee [Comissão de Ética e Bem-Estar Animal (CEBEA)], under protocols described in each published source.

### Data Collection

A dataset that included general information (e.g., author name), qualifying (e.g., sex, level of intake, and block), and necessary quantitative data of body composition and intake was gathered for this study. Data from individual animals were obtained from six comparative slaughter studies evaluating growing Saanen goats of different sexes from 5 to 45 kg of BW (5, 9–13). Animal's age ranged from 20 to 432 days for all sexes. All six studies adopted a randomized block as experimental design, where each block was composed of three pair-fed goats within sex randomly allocated to one of three levels of intake (*ad libitum*; moderate restriction, 25 or 30% of feed restriction; and maintenance level, 50 or 60% of feed restriction). The daily intake of the restricted-fed goats within a block was determined by the dry matter intake (DMI) of the goat fed *ad libitum* within the same block on the previous day. The crude protein (CP) and metabolizable energy contents of solid diets fed ranged from 137 to 175 g/kg DM and 2.4 to 2.7 Mcal/kg DM, respectively; CP of milk ranged from 283 to 285 g/kg DM. Body and diet protein contents were obtained by N analysis via Dumas combustion using LECO FP-528LC (14).

The net protein requirement for maintenance (NP_M_) was calculated using the comparative slaughter technique (6) and the N balance method as described below. In both approaches, the NP_M_ was estimated as the intercept of the linear regression of N retained against N intake (Equation 1) multiplied by 6.25.

(1) $$\begin{array}{l} {\text{N retained}}_{\text{ijk}}={\text{a}}_{\text{i}}+{\text{b}}_{\text{i}}\times {\text{Nintake}}_{\text{ijk}}+{\text{s}}_{\text{j}}+{\text{z}}_{\text{k}\left(\text{j}\right)}+{\text{e}}_{\text{ijkl}} \end{array}$$

N retained_ijk_ is the dependent variable for the *l*^*th*^ animal of the *i*^*th*^ sex in the *j*^*th*^ study in the *k*^*th*^ block; Nintake_ijk_ is the independent variable for the *l*^*th*^ animal of the *i*^*th*^ sex in the *j*^*th*^ study in the *k*^*th*^ block; a_j_ and b_j_ are the parameters to be estimated for each of the *i* = 1, 2, 3 sexes; s_j_ is the random effect of the *j*^*th*^ study ~ *N* (0, $\sigma_{s}^{2}$); z_k(j)_ is the effect of *k*^*th*^ block nested in study *j*^*th*^; and e_ijkl_ is the residual error ~*N* (0, $\sigma_{e}^{2}$).

### Comparative Slaughter Technique

The daily N retained was estimated using the differences between the final body N content at slaughter and the initial body N content. The initial body N content was calculated as follows: (1) initial empty BW (EBW) of the animals was predicted from initial BW using the equation described by Souza et al. (3); and (2) initial body N was predicted from initial EBW across all studies using allometric equations for body protein described by Souza et al. (3) using the animals fed *ad libitum* that were included herein. The summary statistics of the main variables of the dataset by sex were presented (Table 1).

**Table 1:** Summary of descriptive statistics of body composition and intake of Saanen goats used in the comparative slaughter technique.

**Variables**	***n*^a^**	**Mean**	**SD**	**Range**
**BW (kg)**				
All animals	185	27.4	12.5	6.2–51.0
Castrated male	62	27.6	11.0	6.2–47.4
Intact male	80	25.8	13.5	8.0–51.0
Female	43	30.2	12.5	8.4–46.0
**EBW^b^ (kg)**				
All animals	185	22.6	10.9	4.1–41.7
Castrated male	62	22.5	9.50	4.1–39.7
Intact male	80	21.0	11.6	5.1–41.7
Female	43	25.4	11.3	6.6–40.4
**ADG^c^ (g/day)**				
All animals	185	93.7	66.8	−18.8 to 264
Castrated male	62	91.2	71.2	−16.8 to 259
Intact male	80	112	66.5	−13.6 to 264
Female	43	64.0	48.1	−18.8 to 162
**DMI^d^ (g/day)**				
All animals	185	604	364	104.7–1,528
Castrated male	62	701	376	127.6–1,440
Intact male	80	530	362	104.7–1,528
Female	43	604	325	130.9–1,287
**CPI^e^ (g/day)**				
All animals	185	95.2	46.7	23.8–209
Castrated male	62	106	49.3	26.7–205
Intact male	80	87.6	45.5	23.8–209
Female	43	93.4	43.0	29.1–193
**Protein retained in tissue (g/day)**				
All animals	185	13.5	11.0	−12.6 to 53.1
Castrated male	62	14.5	11.7	−3.8 to 41.1
Intact male	80	15.4	11.3	−12.6 to 53.1
Female	43	8.30	7.20	−6.8 to 24.9

a *Number of records*.

b *Empty body weight at slaughter*.

c *Average daily gain*.

d *Dry matter intake*.

e *Crude protein intake*.

For estimating NP_M_, using the comparative slaughter technique, we used data of 185 dairy goats (62 castrated males, 80 intact males, and 43 females). A linear regression of N retained in the daily gain (g of N/kg^0.75^ BW and g of N/kg^0.75^ EBW) on N intake (g of N/kg^0.75^ BW and g of N/kg^0.75^ EBW) was used to calculate the net N requirement for maintenance (Equation 1). The intercept of the regression (i.e., a_i_ parameter) was assumed to be the endogenous and metabolic losses of N, which when multiplied by 6.25 is assumed to be the NP_M_.

### N Balance Method

For estimating NP_M_ using the N balance method, we used data of 136 dairy goats obtained from digestibility trials (Table 2; 43 castrated males, 59 intact males, and 34 females). The feed intake and feed refusals were recorded; and feces and urine were collected for a minimum period of 5 days after an adaptation period as detailed in the published sources. We adopted 0.018 g N/kg^0.75^ BW to dermal losses (15). The N retained in this method was obtained as the difference between N intake and N excreted (sum of fecal, urinary, and dermal N). Similar to the comparative slaughter technique, the intercept of the regression of N retained on N intake was assumed to be the endogenous and metabolic losses of N, which when multiplied by 6.25 is assumed to be the NP_M_ (Equation 1).

**Table 2:** Summary of descriptive statistics of N balance in Saanen goats used in this study.

**Variables**	***n*^a^**	**Mean**	**SD**	**Range**
**BW^b^ (kg)**				
All animals	136	27.2	10.0	7.7–42.1
Castrated male	43	27.7	8.25	7.7–40.2
Intact male	59	24.6	11.5	7.9–42.1
Female	34	31.0	8.00	8.0–39.6
**DMI^c^ (g/day)**				
All animals	136	724	339	43.7–1,672
Castrated male	43	730	339	79.8–1,299
Intact male	59	710	358	64.7–1,672
Female	34	738	310	43.7–1,339
**N intake (g/day)**				
All animals	136	18.1	8.50	1.11–37.6
Castrated male	43	19.4	9.68	2.02–37.6
Intact male	59	17.6	8.27	1.64–36.8
Female	34	17.5	7.33	1.11–32.2
**N feces (g/day)**				
All animals	136	5.42	3.05	0.430–13.3
Castrated male	43	6.31	3.46	0.599–13.3
Intact male	59	4.71	2.88	0.430–12.2
Female	34	5.53	2.52	0.472–11.7
**N urine (g/day)**				
All animals	136	8.39	5.10	0.559–24.9
Castrated male	43	9.87	5.22	0.934–24.9
Intact male	59	7.63	5.39	0.740–21.5
Female	34	7.87	3.90	0.559–14.2
**CP digestibility^d^**				
All animals	136	0.70	0.0846	0.49–0.91
Castrated male	43	0.68	0.0552	0.57–0.81
Intact male	59	0.72	0.107	0.56–0.82
Female	34	0.68	0.0590	0.49–0.91
**DM digestibility^e^**				
All animals	136	0.70	0.0586	0.53–0.88
Castrated male	43	0.71	0.0524	0.56–0.88
Intact male	59	0.69	0.0640	0.53–0.82
Female	34	0.71	0.0542	0.60–0.83

a *Number of records*.

b *Body weight during the digestibility period*.

c *Dry matter intake*.

d *Crude protein digestibility coefficient (g/kg of DM)*.

e *Dry matter digestibility coefficient (g/kg of DM)*.

### Metabolizable Protein Requirements

The metabolizable protein requirement for maintenance (MP_M_) was estimated from the regression of retained protein (g/kg^0.75^ BW and g/kg^0.75^ EBW; calculated using the comparative slaughter technique) against the metabolizable protein intake (MPI; Equation 2). The MPI (g/kg^0.75^ BW or g/kg^0.75^ EBW) was calculated based on the true digestible microbial protein synthesis (MPS) plus the digestible rumen non-degradable protein intake. The MPS was estimated based on the results of a complementary study developed by (16) using the following equation: [MPS (g/day) = 18.13 + 12.48 × MEI (Mcal/day)]. The metabolizable energy intake (MEI; Mcal/day) of each animal was calculated on a dataset including gross energy intake, total energy losses from feces, urine, and gaseous products (17). True fraction and digestibility of microbial protein were both considered 80% (18). The rumen non-degradable protein intake was estimated using the feed composition reported by NRC (1), and its intestinal digestibility adopted was 80%. The MP_M_ was obtained by assuming the retained protein as equal to 0.

(2) $$\begin{array}{l} {\text{Protein retained}}_{\text{ijk}}={\text{a}}_{\text{i}}+{\text{b}}_{\text{i}}\times {\text{MPI}}_{\text{ijk}}+{\text{s}}_{\text{j}}+{\text{z}}_{\text{k}\left(\text{j}\right)}+{\text{e}}_{\text{ijkl}} \end{array}$$

Protein retained_ijk_ is the dependent variable for the *l*^*th*^ animal of the *i*^*th*^ sex in the *j*^*th*^ study in the *k*^*th*^ block; MPI_ijk_ is MPI, the independent variable for the *l*^*th*^ animal of the *i*^*th*^ sex in the *j*^*th*^ study in the *k*^*th*^ block; a_j_ and b_j_ are the parameters to be estimated for each of the *i* = 1, 2, 3 sexes; s_j_ is the random effect of the *j*^*th*^ study ~ *N* (0, $\sigma_{s}^{2}$); z_k(j)_ is the effect of *k*^*th*^ block nested in study *j*^*th*^; and e_ijkl_ is the residual error ~ *N* (0, $\sigma_{e}^{2}$).

### Efficiencies of Metabolizable Protein Use

With the use of the results of the comparative slaughter technique, the efficiency of metabolizable protein use for maintenance (k_PM_) was computed as NP_M_/MP_M_. The efficiency of metabolizable use for gain (k_PG_) was estimated using the equation of daily protein retained calculated in the comparative slaughter technique against daily MPI above maintenance. For this calculation, we used the MP_M_ estimated using Equation (2). This regression was set with an intercept equal to 0.

### Statistical Analysis

Statistical analysis in all models was performed using the MIXED procedure of SAS (9.4) software. A mixed model was used assuming sex (i.e., castrated male, intact male, and female) as a fixed effect, and the effect of block nested in study and sex as a random effect. Statistical significance was declared at *P* < 0.10. The slopes and intercepts of each equation were estimated using the ESTIMATE statement of the MIXED procedure in SAS.

The general statistical model used was as follows:

(3) $$\begin{array}{l} {\text{Y}}_{\text{ijk}}={\text{a}}_{0\text{i}}+{\text{a}}_{1}{\text{X}}_{\text{ijk}}+{\text{s}}_{\text{j}}+{\text{z}}_{\text{k}\left(\text{j}\right)}+{\text{e}}_{\text{ijkl}} \end{array}$$

where Y_ijk_ is the dependent variable for the *l*^*th*^ animal of the *i*^*th*^ sex in the *j*^*th*^ study in the *k*^*th*^ block; X_ijk_ is the independent variable for the *l*^*th*^ animal of the *i*^*th*^ sex in the *j*^*th*^ study in the *k*^*th*^ block; a_0i_ and a_1i_ are the parameters to be estimated for each of the *i* = 1, 2, 3 sexes; s_j_ is the random effect of the *j*^*th*^ study ~ *N* (0, $\sigma_{s}^{2}$); z_k(j)_ is the effect of *k*^*th*^ block nested in study *j*^*th*^; e_ijkl_ is the residual error ~*N* (0, $\sigma_{e}^{2}$).

Three CONTRAST statements were applied to conduct pairwise comparisons of sex. Furthermore, three CONTRAST statements were applied to conduct pairwise comparisons when the interaction between sex and BW or EBW was found to be significant, indicating that at least two slopes differed between sexes (19). Outliers were removed when their normalized residuals were >|3|. For the comparative slaughter technique, five data points were removed (two castrated males, two intact males, and one female). For the N balance method, five data points related to different animals were removed (two castrated males, one intact male, and two females). For estimating the MP_M_, two outliers were removed (one castrated male and one intact male).

Monte Carlo simulations were performed to obtain the lower 90% confidence interval (LCI) and upper 90% confidence interval (UCI) of the MP_M_ estimates. We calculated 10,000 simulated values for each of these protein requirements with a multivariate normal distribution for the parameters and error estimates, using the algorithm reported by Fan et al. (20).

## Results and Discussion

### Net Protein Requirements for Maintenance

**Figure 1:**
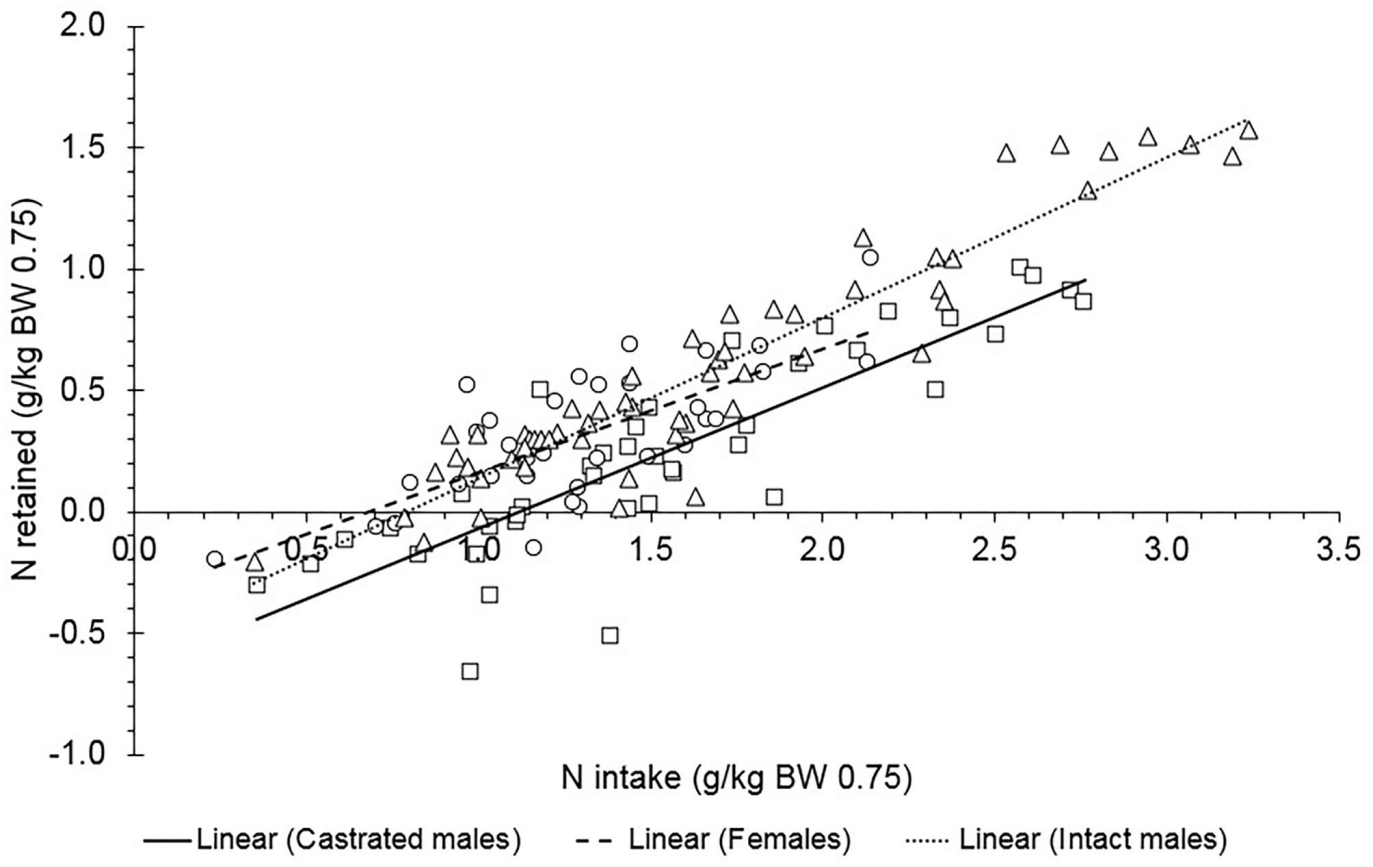
Relationship between daily N retained (g/kg^0.75^ BW) and daily N intake (g/kg^0.75^ BW) of Saanen goats of different sexes using the comparative slaughter technique. For all animals: N retained = −0.197 (±0.0300) + 0.292 (±0.0179) × N intake. The estimated block nested to study variances ($\sigma_{\text{b}:\text{s}}^{2}$) and the residual variances ($\sigma_{\text{e}}^{2}$) were 0.0109 and 0.00655, respectively. The parameters of the equation did not differ between sexes (*P* = 0.67). The observations were adjusted for the study effect.

Using the comparative slaughter technique, we evaluated the relationship between N intake (g/kg^0.75^ BW) and N retained in tissues (g/kg^0.75^ BW) in Saanen goats (Figure 1; Equations 4–7); *n* = 180, $\sigma_{\text{b}:\text{s}}^{2}$ = 0.0109, $\sigma_{\text{e}}^{2}$ = 0.00655). The NP_M_ (i.e., the intercept of this regression multiplied by 6.25) did not differ between sexes (*P* = 0.67), and the overall value was 197 mg of N/kg^0.75^ BW (at N intake = 0), which corresponds to a NP_M_ of 1.23 g/kg^0.75^ BW.

(4) $$\begin{array}{l} \text{Castrated male}:\text{ N retained}=-0.233\left(\pm 0.0487\right)\\ \;+0.293\left(\pm 0.0264\right)\times \text{N intake} \end{array}$$

(5) $$\begin{array}{l} \text{Intact male}:\text{ N retained}=-0.182\left(\pm 0.0431\right)\\ \;+0.334\left(\pm 0.0241\right)\times \text{N intake} \end{array}$$

(6) $$\begin{array}{l} \text{Female}:\text{ N retained}=-0.176\left(\pm 0.0622\right)\\ \;+0.249\left(\pm 0.0401\right)\times \text{N intake} \end{array}$$

(7) $$\begin{array}{l} \text{All sexes}:\text{ N retained}=-0.197\left(\pm 0.0300\right)\\ \;+0.292\left(\pm 0.0179\right)\times \text{N intake} \end{array}$$

When this equation was scaled by metabolic EBW, the NP_M_ (i.e., the intercept of this regression) also did not differ between sexes (*P* = 0.61). We presented the relationship between N intake (g/kg^0.75^ EBW) and N retained (g/kg^0.75^ EBW) in Saanen goats (Equations 8–11; *n* = 180, $\sigma_{\text{b}:\text{s}}^{2}$ = 0.0144, $\sigma_{\text{e}}^{2}$ = 0.00916). The overall value of NP_M_ (i.e., the intercept of this regression multiplied by 6.25) was 1.46 g/kg^0.75^ EBW.

(8) $$\begin{array}{l} \text{Castrated male}:\text{ N retained}=-0.281\left(\pm 0.0576\right)\\ \;+0.298\left(\pm 0.0268\right)\times \text{N intake} \end{array}$$

(9) $$\begin{array}{l} \text{Intact male}:\text{ N retained}=-0.210\left(\pm 0.0507\right)\\ \;+0.333\left(\pm 0.0246\right)\times \text{N intake} \end{array}$$

(10) $$\begin{array}{l} \text{Female}:\text{ N retained}=-0.211\left(\pm 0.0738\right)\\ \;+0.255\left(\pm 0.0417\right)\times \text{N intake} \end{array}$$

(11) $$\begin{array}{l} \text{All sexes}:\text{ N retained}=-0.234\left(\pm 0.0355\right)\\ \;+0.295\left(\pm 0.0184\right)\times \text{N intake} \end{array}$$

**Figure 2:**
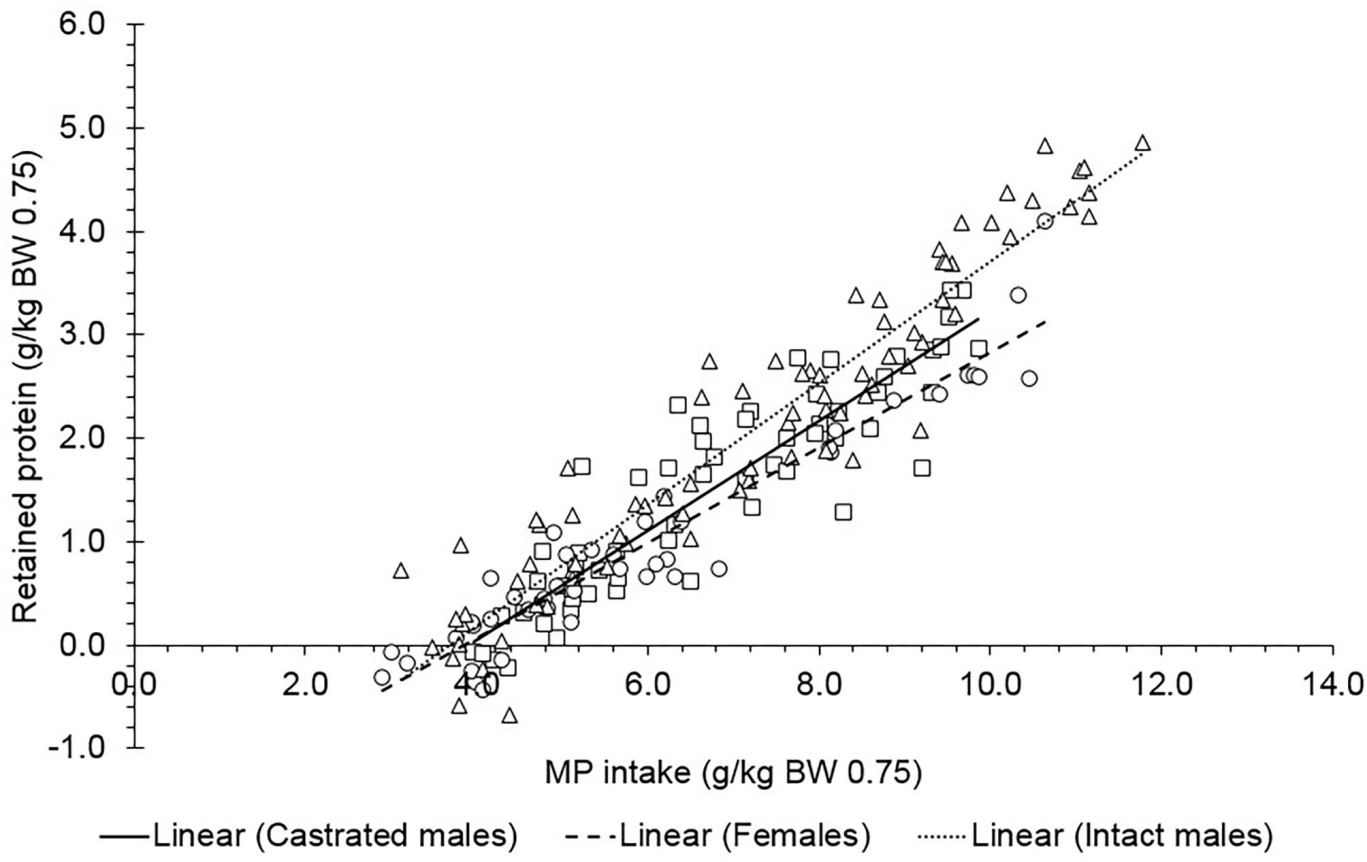
Relationship between daily N retained (g/kg^0.75^ BW) and daily N intake (g/kg^0.75^ BW) of Saanen goats of different sexes using the N balance method. For all animals: N retained = −0.509 (±0.0817) + 0.584 (±0.0519) × N intake. The estimated block nested to study variances ($\sigma_{\text{b}:\text{s}}^{2}$) and the residual variances ($\sigma_{\text{e}}^{2}$) were 0.0503 and 0.0482, respectively. The parameters of the equation did not differ between sexes (*P* = 0.38). The observations were adjusted for the study effect.

Using the N balance approach, we evaluated a relationship between N intake (g/kg^0.75^ BW) and N retained in N Balance (g/kg^0.75^ BW) in Saanen goats (Figure 2; Equations 12–15); *n* = 131, $\sigma_{\text{b}:\text{s}}^{2}$ = 0.0503, $\sigma_{\text{e}}^{2}$ = 0.0482). The NP_M_ (i.e., the intercept of this regression multiplied by 6.25) also did not differ between sexes (*P* = 0.38), and the overall value was 509 mg of N/kg^0.75^ BW (at N intake = 0), which corresponds to a NP_M_ of 3.18 g/kg^0.75^ BW.

(12) $$\begin{array}{l} \text{Castrated male}:\text{ N retained}=-0.653\left(\pm 0.135\right)\\ \;+0.582\left(\pm 0.0773\right)\times \text{N intake} \end{array}$$

(13) $$\begin{array}{l} \text{Intact male}:\text{N retained}=-0.525\left(\pm 0.111\right)\\ \;+0.662\left(\pm 0.0596\right)\times \text{N intake} \end{array}$$

(14) $$\begin{array}{l} \text{Female}:\text{ N retained}=-0.348\left(\pm 0.172\right)\\ \;+0.508\left(\pm 0.121\right)\times \text{N intake} \end{array}$$

(15) $$\begin{array}{l} \text{All sexes}:\text{N retained}=-0.509\left(\pm 0.0817\right)\\ \;+0.584\left(\pm 0.0519\right)\times \text{N intake} \end{array}$$

When this equation was scaled by metabolic EBW, the NP_M_ (i.e., the intercept of this regression) also did not differ between sexes (*P* = 0.36). We presented the relationship between N intake (g/kg^0.75^ EBW) and N retained (g/kg^0.75^ EBW) in Saanen goats (Equations 16–19; *n* = 131, $\sigma_{\text{b}:\text{s}}^{2}$ = 0.0669, $\sigma_{\text{e}}^{2}$ = 0.0647). The overall value of NP_M_ (i.e., the intercept of this regression multiplied by 6.25) was 3.76 g/kg^0.75^ EBW.

(16) $$\begin{array}{l} \text{Castrated male}:\text{ N retained}=-0.779\left(\pm 0.159\right)\\ \;+0.591\left(\pm 0.0786\right)\times \text{N intake} \end{array}$$

(17) $$\begin{array}{l} \text{Intact male}:\text{ N retained}=-0.616\left(\pm 0.124\right)\\ \;+0.664\left(\pm 0.0557\right)\times \text{N intake} \end{array}$$

(18) $$\begin{array}{l} \text{Female}:\text{ N retained}=-0.411\left(\pm 0.202\right)\\ \;+0.514\left(\pm 0.123\right)\times \text{N intake} \end{array}$$

(19) $$\begin{array}{l} \text{All sexes}:\text{ N retained}=-0.602\left(\pm 0.0952\right)\\ \;+0.589\left(\pm 0.0522\right)\times \text{N intake} \end{array}$$

The estimates of NP_M_ obtained using the comparative slaughter technique were ~50% lower than those obtained using the N balance method. It has been reported that N balance overestimates the values and results in a greater variation for the protein requirements for maintenance (5, 7). The overall value using the comparative slaughter technique was lower than that reported by AFRC [(4); 2.19 g/kg^0.75^ BW of NP_M_], where the system adopted a k_PM_ of 1.0 to maintenance. The daily requirement reported by AFRC is based on the sum of basal endogenous losses of N, considering the urinary, fecal, and dermal N.

In both approaches, sex did not affect the NP_M_. The digestibility coefficients for CP (g/kg of DM) calculated using the N balance dataset presented a mean value of 0.70 for all animals. Although females present less body protein than males in dairy goats at a given BW (3), those differences possibly did not affect the protein requirements for maintenance in this study. Considering the mature weight previously estimated for Saanen goats (21) where females reach chemical maturity at lower BW (26 kg EBW) than castrated males (34.9 kg EBW) and intact males (42.6 kg EBW), we noted that all intact males evaluated in this study did not reach maturity, although few castrated males and females reached mature weight. We suspect that no effect was observed on the NP_M_ because, in general, all animals were in the growth phase (animal's age ranged from 20 to 432 days for all sexes). The body protein content reaches a plateau close to maturity, and the notable changes in body composition related to sex are consequences of the increase in fat deposition instead of protein synthesis in dairy goats (21). Possibly, we did not find a remarkable difference in the body protein to affect the protein requirements for the maintenance of the dairy goats of different sexes evaluated herein.

The NP_M_ obtained using the comparative slaughter technique is similar to the values obtained by the independent studies used in this dataset, as expected, in a study evaluating goat kids [1.32 g/kg^0.75^ BW in Saanen kids from 5 to 20 kg of BW; (9)], as well as in late growth goats [1.46 g/kg^0.75^ BW in Saanen goats from 30 to 45 kg BW; (5)]. The age seems not to affect the protein requirements for maintenance, similar to that previously described in pregnant dairy goats [1.38 g/kg^0.75^ EBW; (7)].

Comparing our results with other genotypes, the overall value of NP_M_ obtained herein (1.23 g/kg^0.75^ BW) is lower than the NP_M_ obtained in an individual study for Boer goats when also using the comparative slaughter technique [2.04 g/kg^0.75^ of BW; (22)]. Considering these differences between genotype reported in NRC (1), the MP_M_ estimated in Angora goats (3.35 g/kg^0.75^ BW) is greater than the estimates in meat, dairy, and indigenous goats [3.07 g/kg^0.75^ BW; (23, 24)]. Differences in body composition between dairy, meat, or fiber goats may affect the MP_M_ of these animals. Although dairy goats present a heavier gastrointestinal tract than meat goats (25), they present lighter carcass as a consequence of less muscular growth than meat goats at the same age. This is a consequence of the genotypes selected for milk production. Based on that, we could infer that less body protein in the carcass of dairy biotype supports lower body protein losses and, consequently, lower protein requirements for maintenance in dairy goats compared with meat goats.

The remarkable difference between the methods used herein for estimating the protein requirements for maintenance raises previous studies that reported that N balance may overestimate the NP_M_ in metabolism trials. Our results agree with other studies that also reported that N balance can lead to biases of overestimation of N retention (5, 26). Estimates of NP_M_ by the comparative slaughter technique yielded more precise equations than those obtained from the N balance data, which can be verified by the dispersion of the points in the graphs presented herein and in the residual analysis. Similarly, in pregnant goats, a low error was also reported using the comparative slaughter technique, where this approach possibly gave a more reliable reference value in mature goats (7). Slaughter studies are costly, but they indicate a more reliable measure of protein retention (2). The protein retention measured under slaughter methods is directly calculated, where representative samples of body tissues are obtained. Although the N balance trials used in this dataset described an adequate measurement of fecal and urine excretion including urine acidification, it is still a short-term experiment, and it is possibly more susceptible to errors in sample processing and analysis (2). The overestimated losses in N balance may improperly overestimate the protein requirements for maintenance. Additionally, little is known about the metabolic process' influence on N recycling in the body, since short-term regulation as ureagenesis can be reflected in a variation that is not clearly understood (27). Body proteins constantly undergo breakdown and re-synthesis, but these aspects remain unclarified (27). Losses of N during balance trials possible occur through routes that are not considered. In addition, the microbial metabolic activity in the ruminant metabolism and its effect on subsequent processes in the deposition of N in body tissues make the study of N metabolism in ruminants challenging compared with non-ruminants (2).

### Metabolizable Protein Requirements and Efficiencies of Protein Use

**Figure 3:**
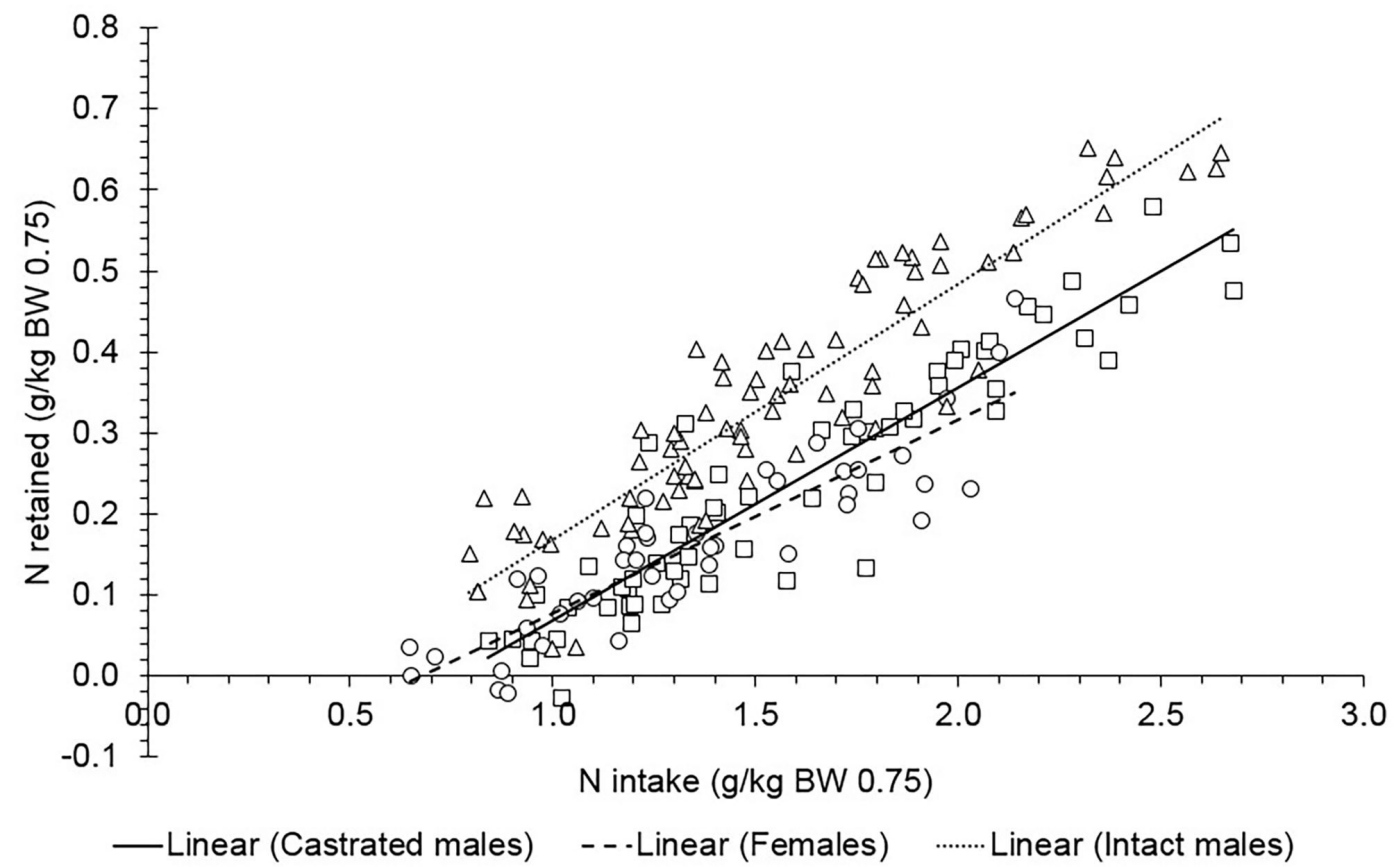
Relationship between daily protein retained (g/kg^0.75^ BW) and daily metabolizable protein (MP) intake (g/kg^0.75^ BW) of Saanen goats of different sexes. For all animals: protein retained = −1.98 (±0.154) + 0.525 (±0.0220) × MPI. The estimated block nested to study variances ($\sigma_{\text{b}:\text{s}}^{2}$) and the residual variances ($\sigma_{\text{e}}^{2}$) were 0.0891 and 0.212, respectively. The parameters of the equation did not differ between sexes (*P* = 0.55). The observations were adjusted for the study effect.

The MP_M_ did not differ between sexes (*P* = 0.557). We presented the relationship between daily protein retained obtained in the comparative slaughter technique approach (g/kg^0.75^ BW) and the daily MPI (g/kg^0.75^ BW) in Saanen goats (Figure 3; Equations 20–23; *n* = 183; $\sigma_{\text{b}:\text{s}}^{2}$ = 0.0891, $\sigma_{\text{e}}^{2}$ = 0.212). The overall value of MP_M_ was 3.8 g/kg^0.75^ BW. The LCI and UCI for MP_M_ were 3.10 and 4.38 g/kg^0.75^ BW, respectively.

(20) $$\begin{array}{l} \text{Castrated male}:\text{ Protein retained}=-2.055\left(\pm 0.288\right)\\ \;+0.528\left(\pm 0.0409\right)\times \text{MPI} \end{array}$$

(21) $$\begin{array}{l} \text{Intact male}:\text{ Protein retained}=-2.164\left(\pm 0.231\right)\\ \;+0.588\left(\pm 0.0299\right)\times \text{MPI} \end{array}$$

(22) $$\begin{array}{l} \text{Female}:\text{ Protein retained}=-1.775\left(\pm 0.278\right)\\ \;+0.460\left(\pm 0.0424\right)\times \text{MPI} \end{array}$$

(23) $$\begin{array}{l} \text{All sexes}:\text{ Protein retained}=-1.98\left(\pm 0.154\right)\\ \;+0.525\left(\pm 0.0220\right)\times \text{MPI} \end{array}$$

When this equation was scaled by metabolic EBW, the MP_M_ (i.e., the intercept of this regression) also did not differ between sexes (*P* = 0.47). We presented the relationship between daily protein retained (g/kg^0.75^ EBW) and daily MPI (g/kg^0.75^ EBW) in Saanen goats (Equations 24–27; *n* = 183; $\sigma_{\text{b}:\text{s}}^{2}$ = 0.119, $\sigma_{\text{e}}^{2}$ = 0.292). The overall value of MP_M_ was 4.4 g/kg^0.75^ EBW. The LCI and UCI for MP_M_ were 3.60 and 5.10 g/kg^0.75^ EBW, respectively.

(24) $$\begin{array}{l} \text{Castrated male}:\text{ Protein retained}=-2.499\left(\pm 0.338\right)\\ \;+0.539\left(\pm 0.0411\right)\times \text{MPI} \end{array}$$

(25) $$\begin{array}{l} \text{Intact male}:\text{ Protein retained}=-2.466\left(\pm 0.269\right)\\ \;+0.582\left(\pm 0.0301\right)\times \text{MPI} \end{array}$$

(26) $$\begin{array}{l} \text{Female}:\text{ Protein retained}=-2.006\left(\pm 0.323\right)\\ \;+0.453\left(\pm 0.0428\right)\times \text{MPI} \end{array}$$

(27) $$\begin{array}{l} \text{All sexes}:\text{ Protein retained}=-2.322\left(\pm 0.180\right)\\ \;+0.525\left(\pm 0.0222\right)\times \text{MPI} \end{array}$$

With the use of the relationship between NP_M_/MP_M_, the calculated k_PM_ was 0.33 for all sexes (the LCI and UCI were 0.28 and 0.40, respectively).

The MP_M_ estimated in this study was similar to that recommended by NRC (1) of 3.07 g/kg MP_M_ kg^0.75^ BW. However, there is a noticeable difference between the k_PM_ estimated in our study and the ones reported by the feeding systems. The k_PM_ computed herein varied from 0.28 to 0.4, whereas values of 0.67 and 1.0 are reported by the most applied feeding systems (1, 4). The NRC (1) refers k_PM_ as 1.0. According to this feeding system (1), the k_PM_ value must be evaluated under distinct situations, for instance, in the well-fed state, or under low nutrient intake with tissue mobilization. To our knowledge, considering 100% efficiency is overestimated. Our findings suggest that knowledge about MP efficiency still deserves further attention, mainly about the estimative of MP and the range of MP levels in different situations.

When applying the equation of daily protein retained against daily MPI above maintenance (g/kg^0.75^ EBW), the equation did not differ between sexes (*P* = 0.82) and the overall k_PG_ was 0.52 in Saanen goats (Equations 28–31; *n* = 183; $\sigma_{\text{b}:\text{s}}^{2}$ = 0.119, $\sigma_{\text{e}}^{2}$ = 0.292). The value of k_PG_ for castrated males, intact males, and females was 0.54 (±0.0411), 0.58 (±0.0301), and 0.45 (±0.0428), respectively.

(28) $$\begin{array}{l} \text{Castrated male}:\text{ Protein retained}=0.539\left(\pm 0.0411\right)\times \text{MPI} \end{array}$$

(29) $$\begin{array}{l} \text{Intact male}:\text{ Protein retained}=0.582\left(\pm 0.0301\right)\times \text{MPI} \end{array}$$

(30) $$\begin{array}{l} \text{Female}:\text{ Protein retained}=0.453\left(\pm 0.0428\right)\times \text{MPI} \end{array}$$

(31) $$\begin{array}{l} \text{All sexes}:\text{ Protein retained}=0.525\left(\pm 0.0222\right)\times \text{MPI} \end{array}$$

The overall k_PG_ calculated (0.52 ± 0.0222) was close to the value reported to goats in AFRC (4) (0.59) but lower than that adopted by SCA (28) and NRC (1), in which both adopted 0.70. This value was also close to that of recent studies in dairy cows by Castro et al. (29) of 0.51. Because the differences in protein content in tissue gain possibly affect this efficiency for growth (1), we would expect different values of k_PG_ between sexes. Although we presented the overall k_PG_ and the variation of results did not allow a difference between sexes, we opted for reporting k_PG_ for each sex, considering that nutritionists may strategically adopt one or other in different situations of animal production: castrated males, 0.54 (±0.0411); intact males, 0.58 (±0.0301); and females, 0.45 (±0.0428).

In bovines, feeding low protein diets has been discussed in the last years, where no differences in animal performance among Nellore bulls fed diets containing 10, 12, or 14% CP were detected (30). The main interest in feeding diets with less protein content is that it can reduce N input, improving N utilization efficiency, thereby reducing the environmental impact caused by N losses from manure (31). Our results of NP_M_ suggest that these requirements for growing dairy goats could be lower than those preconized by the current feeding systems (1).

In conclusion, the current study evaluated the protein requirements for maintenance and efficiency of metabolizable protein use in growing Saanen goats of different sexes, using both the comparative slaughter technique and N balance method, under a meta-analytical approach. Based on our results, we suggest that there is no evidence that sex affects the protein requirements for maintenance and efficiencies of protein use. The equations reported herein may improve the accuracy of protein requirements values adopted to dairy goats, thereby reducing the cost of the diets, as well as the environmental and social impacts of animal production.

## Data Availability Statement

The data that support the findings of this study are available from the corresponding author upon reasonable request.

## Ethics Statement

All procedures used in the individual studies were followed by the University's Animal Care Committee (Comissão de Ética e Bem-Estar Animal – CEBEA), under protocols described in each one of the published sources.

## Author Contributions

AS, JV, MF, AA, and IT: data curation, conceptualization, methodology, writing—original draft, review, and editing. KR and IT: funding acquisition, conceptualization, supervision, and project administration. All authors contributed to the article and approved the submitted version.

## Conflict of Interest

The authors declare that the research was conducted in the absence of any commercial or financial relationships that could be construed as a potential conflict of interest.
